# Study on the Effects of National Volume‐Based Procurement of Chemical Drugs on Chinese Patent Medicines: Lipid‐Lowering Drugs as an Example

**DOI:** 10.1002/hcs2.70003

**Published:** 2025-02-22

**Authors:** Zhao Yang, Xiao Han, Pei Liang, Xiaoting Zhao, Qiyun Zhu, Hui Ye, Chao Yang, Bin Jiang

**Affiliations:** ^1^ Center for Digital Health and Artificial Intelligence Peking University First Hospital Beijing China; ^2^ Research Center of Public Policy Peking University Beijing China; ^3^ School of Public Health Shanghai Jiaotong University Shanghai China; ^4^ Women and Children's Hospital of Chongqing Medical University Chongqing Health Center for Women and Children Chongqing China; ^5^ School of Pharmaceutical Sciences Peking University Beijing China; ^6^ Revelle College University of California San Diego San Diego California USA; ^7^ TCM‐Integrated TCM & Western Medicine Dept Peking University First Hospital Beijing China; ^8^ Renal Division, Department of Medicine Peking University First Hospital, Peking University Institute of Nephrology Beijing China

**Keywords:** Chinese patent medicine, health policy, lipid‐lowering drugs, national volume‐based procurement

## Abstract

**Background:**

Atherosclerotic cardiovascular disease remains the leading cause of death worldwide. This study aims to explore the impact of national volume‐based procurement (NVBP) on Chinese patent medicines and provide evidence for improving policies and promoting rational drug use.

**Methods:**

The study was based on data from the China National Health Insurance Agency that spanned January 2019 to December 2020. Descriptive analysis was conducted using volume and expenditure as variables. Interrupted time series analysis was applied to further analyze Chinese patent medicines.

**Results:**

The unit prices of atorvastatin and rosuvastatin decreased by 25%–96%, whereas the prices of Zhibitai and Xuezhikang fluctuated slightly. The affordability is measured as the monthly expenditure on treatment divided by the daily wage. After policy implementation, the affordability of atorvastatin and rosuvastatin improved from 0.242 to 0.014 and from 0.247 to 0.019, respectively. The defined daily doses (DDDs) for atorvastatin and rosuvastatin also increased, whereas total expenditures decreased in hospitals of all levels. Both at the national level and at all levels of hospital, the policy had no significant impact on expenditures for Zhibitai and Xuezhikang and their defined daily doses.

**Conclusions:**

The NVBP saved costs in the short term by incorporating high‐quality, widely used lipid‐lowering drugs. Notably, the policy impacted lipid‐lowering chemical drugs, whereas Chinese patent medicines remained largely unaffected. Doctors' use of Chinese patent medicines did not decline, highlighting the clinical specificity of these medicines.

## Graphical abstract

This study analyzes the impact of national volume‐based procurement (NVBP) on lipid‐lowering drugs before and after policy implementation using 24 months of data. The results showed that there was a significant increase in the use of bid‐winning statins, but the policy did not cause an overall change in the use of proprietary Chinese medicines.

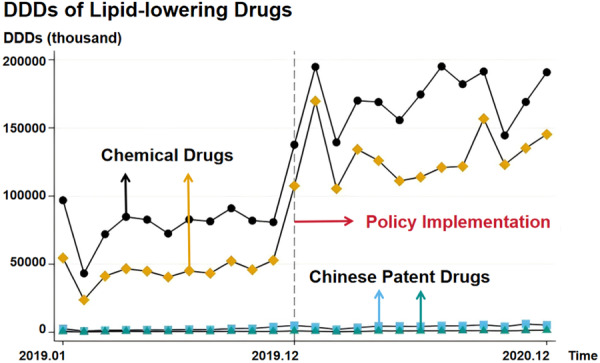

**Abbreviations**
ASCVD: atherosclerotic cardiovascular disease
DDDs: defined daily doses
ITS: interrupted time series analysis
NVBP: national volume‐based procurement

## 1 Introduction

In China, atherosclerotic cardiovascular disease (ASCVD) is the leading cause of death in both urban and rural populations [1]. The prevalence of cardiovascular disease is expected to increase as China's aging population grows, suggesting that the rise in ASCVD and related diseases will add to the social and family burden [2]. Hyperlipidemia, especially low‐density lipoprotein cholesterol (LDL‐C), is a pathogenic risk factor for ASCVD [3]. Improving the awareness, treatment, and control rates of blood lipid abnormalities among the general public and patients with ASCVD is a core strategy for the primary and secondary prevention of ASCVD [4].

High‐dose statins that have been approved for Western populations may not be suitable for Chinese patients. High‐dose statin therapy may pose certain safety concerns. The latest guidelines [5] specify using Chinese patent medicines such as Xuezhikang or Zhibitai as initial lipid‐lowering treatment. These drugs not only lower blood lipid levels but also significantly improve patients' clinical symptoms and alleviate their discomfort [6, 7]. The two drugs are currently the most widely used lipid‐lowering Chinese patent medicines. A large number of studies support the unique advantages of these drugs as part of multi‐component, multi‐channel, and multi‐target treatment strategies [8, 9]. Therefore, we selected Xuezhikang and Zhibitai as representative Chinese patent medicines to study the policy's impact on lipid‐lowering Chinese patent medicines. Xuezhikang is a Chinese lipid‐lowering patent medicine that is refined through the fermentation of special red yeast and contains a variety of natural compound statin ingredients [8, 10]. Zhibitai contains statins, flavonoids, triterpenoids, and organic acids. Triterpenoids and flavonoids are effective in the treatment of atherosclerosis by lowering blood lipids, protecting blood vessels, dilating coronary arteries, improving myocardial function, and lowering blood pressure. In clinical practice, these compounds are more suitable for the physical and disease characteristics of the Chinese population and have a comprehensive lipid‐regulating effect than chemical drugs. In 2018, the National Medical Security Bureau launched the “4 + 7” city drug centralized procurement pilot program, which included four municipalities (Beijing, Shanghai, Tianjin, and Chongqing) and seven key cities (Xi'an, Shenyang, Dalian, Guangzhou, Chengdu, Shenzhen, and Xiamen) in important provinces. In September 2019, the National Healthcare Security Administration and nine other departments issued *Implementation Opinions on the National Volume‐Based Procurement (NVBP) and Expanding the Regional Scope of the Utilization Pilot*, thus initiating the first batch of drug volume procurement. Based on the “4 + 7” city pilot program, the policy was expanded to cover 25 regions nationwide [11]. The National Healthcare Security Administration formulates a nationwide uniform guidebook and supervisory mechanisms, and all provincial regions adhere to a unified drug procurement process and standards. Previous studies have found that the policy of procurement of chemical drugs can simultaneously reduce the prices of bid‐winning drugs, decrease the cost burden of medication on the public, and change the structure of medication used in medical institutions. Studies in Tianjin and Shenzhen have provided valuable insights into the impact of the policy; these investigations indicated that the policy facilitated generic substitution and savings in procurement costs through lower drug prices. However, these studies were often limited to a single city or province. Our study utilizes nationwide data that allow a broader geographical representation and a more comprehensive perspective. Previous studies that have assessed the impact of policies on targeted drugs, antiviral medications, antibiotic drugs, and antihypertensive drugs have suggested a positive effect on drug utilization for these types of medications. However, the performance under the policy of lipid‐lowering drugs, which are essential for cardiovascular disease prevention and treatment, has not been adequately studied. Studies have focused on the impact of policies on chemical drugs such as originator drugs and generics; however, the impact on Chinese patent medicines has not yet been studied. As the national policy continues to advance, the centralized procurement of Chinese patent medicines—an important component of the pharmaceutical industry—has become regularized. The policy orientation and implementation effect of the first batch of centralized procurement of Western medicines holds significant reference value for the formulation and execution of the centralized procurement policy for Chinese patent medicines. The volume‐based procurement of medicine may lead to industry reshuffling and drive changes to corporate strategy and the transformation of enterprises. Chinese patent medicine enterprises must learn from the experience of Western medicine procurement and optimize internal management to swiftly adjust their strategies to achieve sustainable development. The policy reduces the burden on patients by lowering drug prices. Studying the changes in price for Xuezhikang and Zhibitai under this policy can directly reflect the changes in patients' financial burden. The study elucidates the impact of the national procurement policy on the Chinese patent medicine market by investigating price changes, the competitive market landscape, and the accessibility of various lipid‐lowering drugs to patients. This understanding can help predict future market trends to allow the adjustment of market strategies and provide policymakers with a better grasp of the market impact of collection and procurement policy to support more scientific and practical policy formulation. Given the global promotion of Chinese medicine, studying the impact of China's policy on Chinese patent medicine can also provide references and lessons for the international market.

Therefore, focusing on lipid‐lowering medications, this study investigates changes in defined daily doses (DDDs) and expenditures of bid‐winning statins (atorvastatin and rosuvastatin) and non‐purchased Chinese patent medicines (Zhibitai and Xuezhikang) before and after policy implementation based on data from 25 provincial‐level administrative regions in the National Medical Security Bureau database. We aimed to explore the impact of NVBP on Chinese patent medicines in medical institutions and provide scientific evidence for improving policies and promoting rational drug use.

## 2 Materials and Methods

In this study, NVBP policy‐related antihyperlipidemics and their alternative Chinese patent medicines were included for study. Atorvastatin and rosuvastatin are policy‐related drugs. Alternative Chinese patent medicines such as Xuezhikang and Zhibitai were also included. Notably, the pricing mechanism of drugs in China is strictly controlled by national policies and regulatory agencies and does not change over time.

### 2.1 Data Sources

Data on products purchased between January 2019 and December 2020 were extracted from the National Health Insurance Agency. The data set included the procurement records of 1.115 million health facilities in 24 provincial‐level administrative regions and covered a population exceeding 762.0 million.

### 2.2 Outcome Measures

Two outcome measures were included: volume and expenditures. Volume was measured using DDDs, and expenditure data were reported in CNY.

#### 2.2.1 Volume

Volume was measured using DDDs, a method developed by the World Health Organization to compare drug consumption across medicines. DDDs represent the annual consumption of each drug divided by the DDD (the average daily dose of the drug used for primary therapeutic purposes in adults). In this study, the DDD of each medication was determined in accordance with the Guidelines for ATC Classification and DDD Assignment 2021. DDDs can reflect medication dynamics and structure across years. The larger the DDD, the higher the frequency of drug use.

#### 2.2.2 Expenditure Data Reported in CNY

In addition, to elucidate the general changing trend of drug accessibility, we introduced the concept of drug affordability. Multiple dimensions, among which the two most important are availability and affordability, are used to measure drug accessibility [12]. Drug availability can be reflected in drug allocation and drug shortages in medical institutions. The World Health Organization guidelines define drug affordability as the ratio of the cost of treatment courses to the minimum daily wages of national government employees (extra daily wages) [13]. Because China does not calculate extra daily wages, we used the daily per capita disposable income in line with previous research.

In this study, affordability was measured as the ratio of monthly expenditures to the daily wage:

Affordability = cost of monthly expenditure on treatment/daily wage [14].

Affordability reflects residents' ability to pay for drugs; therefore, it is a relevant indicator of people's livelihoods.

### 2.3 Statistical Analysis

Interrupted time series analysis (ITS) is a quasi‐experimental design that allows the collection at multiple time points of measured results and indicators before and after interventions are implemented, compares immediate changes in level and regression slopes in a specific period before and after interventions, and then assesses the effectiveness of intervention measures. Currently, this method is considered the best quasi‐experimental research design for evaluating the long‐term impact of policy interventions.

We utilized the ITS method in this study. The intervention point was set as December 2019. The 12 months from January 2019 to December 2019 and the 12 months from January 2020 to December 2020 were the pre‐ and post‐policy implementation intervals, respectively. We estimated the DDDs and expenditure for the four types of lipid‐lowering drugs and fitted the ITS model.

The construction of the discrete time‐series model was equation (1):

(1) $$Y_{t}=\beta_{0}+\beta_{1}\times \text{Time}+\beta_{2}\times \text{Policy}+\beta_{3}\times \text{Trend}+\varepsilon_{t}$$

where *Y*_t_ was the outcome variable (volume, expenditures), *β*_0_ estimated the baseline level of the outcome variable at the beginning of the observation period, and *β*_1_ estimated the slope before intervention. *β*_2_ estimated the change in level in the period immediately following policy intervention, and *β*_3_ estimated the differences between the pre‐ and post‐intervention slopes. *ε*_t_ was an estimate of the random error at time t.

The Durbin–Watson test was performed to indicate the model's adjustment effect on autocorrelation. A Durbin–Watson *d* value of approximately 2 indicates no autocorrelation. All analyses were performed using Stata SE 16 (Stata Corp. LP, College Station, TX, USA).

## 3 Results

### 3.1 Affordability

This study included four lipid‐lowering drugs: atorvastatin, rosuvastatin, Xuezhikang, and Zhibitai. The total purchase volume was 5349.88 million DDDs, and the total expenditure was 13.998 billion CNY. Atorvastatin and rosuvastatin were included in the national drug centralized procurement policy in 2019, whereas the Chinese patent medicines Xuezhikang and Zhibitai were not included in the centralized procurement category. We analyzed the affordability of the four drugs across various provinces in China to observe the impact of policies on related and alternative drugs.

Table 1 shows that after policy implementation, the unit prices of atorvastatin and rosuvastatin decreased significantly, whereas the unit prices of Xuezhikang and Zhibitai fluctuated slightly. The reduction in the price of 10 mg of atorvastatin ranged from −81.38% to −96.64%, and that of 20 mg of atorvastatin was −90.54%. Rosuvastatin's price dropped from −89.85% to −93.49%. Before centralized procurement, the monthly expenditure for Xuezhikang and Zhibitai was higher than that of atorvastatin and rosuvastatin. After centralized procurement, the monthly expenditure for Xuezhikang was 14.42–37.84 times that of atorvastatin and 15.20–22.71 times that of rosuvastatin; the monthly expenditure for Zhibitai was 22.22–58.34 times that of atorvastatin and 23.44–35.00 times that of rosuvastatin.

**Table 1:** Characteristics of antihyperlipidemic drugs.

Drugs	Manufacturers	Specifications (mg)	Unit price (CNY)		Price change (%)	Monthly expenditure (CNY)	
			Pre	Post		Pre	Post
Atorvastatin	Qilu	10	3.57	0.12	96.64	107.06	3.60
	Xing'an	10	2.60	0.13	95.00	78.00	3.86
	Lepu	10	1.69	0.32	81.07	50.76	9.45
	Lepu	20	5.80	0.55	90.52	87.00	8.23
Rosuvastatin	Hanhui	10	2.94	0.20	93.20	88.21	6.00
	Lek	10	3.50	0.23	93.43	105.00	6.84
	Chia‐Tai	10	2.94	0.30	89.80	88.25	8.96
Zhibitai	Diao Jiuhong	1	4.56	4.54	0.44	136.82	136.23
Xuezhikang	Weixin	1	6.90	7.00	**−**1.45	206.99	210.01

*Note:* Full name of the companies: Qilu: Qilu Pharmaceutical Co. Ltd.

Xing'an: Fujian Xing'an Pharmaceutical Co. Ltd.

Lepu: Zhejiang Lepu Pharmaceutical Co. Ltd.

Hanhui: Hanhui Pharmaceutical Co. Ltd.

Lek: Lek Pharmaceutical and Chemical Company d.d.

Chia‐Tai: Chia Tai Tianqing Pharmaceutical Group Co. Ltd.

Diao Jiuhong: Chengdu Diao Jiuhong Pharmaceutical Factory

Weixin: Beijing Peking University WBL Biotech Co. Ltd.

Table 2 shows the changes in the affordability of the four drugs before and after policy implementation. After implementation, the average affordability of atorvastatin improved from 0.242 to 0.014, and the average affordability of rosuvastatin improved from 0.247 to 0.019. The worst affordability observed among the four drugs was less than 1, signifying that all provinces could still afford the drug; nevertheless, policy implementation still improved affordability.

**Table 2:** Affordability of antihyperlipidemic drugs before and after policy intervention.

Drugs	Pre‐NVBP			Post‐NVBP		
	Min	Mean	Max	Min	Mean	Max
Atorvastatin	0.138	0.242	0.380	0.005	0.014	0.027
Rosuvastatin	0.140	0.247	0.348	0.010	0.019	0.032
Xuezhikang	0.313	0.557	0.746	0.308	0.549	0.735
Zhibitai	0.204	0.363	0.486	0.203	0.361	0.484

### 3.2 National DDDs and Expenditure

**Figure 1:**
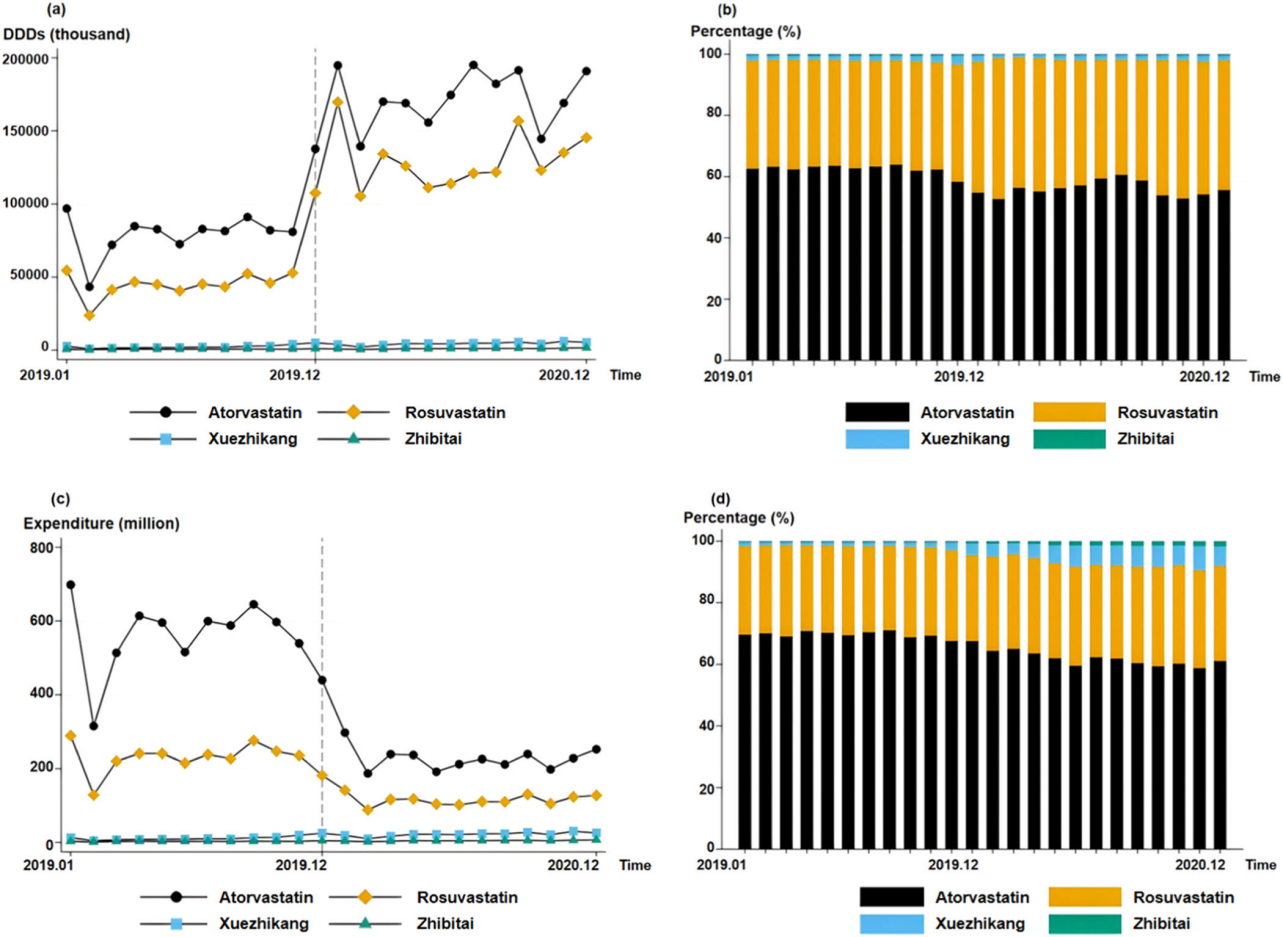
DDDs and expenditure for antihyperlipidemic drugs at the national level. (a) Trend analysis of DDDs for antihyperlipidemic drugs at the national level. (b) Percentage distribution of DDDs for antihyperlipidemic drugs at the national level. (c) Trend analysis of expenditure for antihyperlipidemic drugs at the national level. (d) Percentage distribution of expenditure antihyperlipidemic drugs at the national level.

Figure 1 shows the DDDs and expenditures of the four drugs nationwide. After entering the centralized procurement range, the dosages of atorvastatin and rosuvastatin increased while the overall costs decreased. Specifically, the DDDs of atorvastatin increased from 1008.0 million in 2019 to 2076.2 million (+106.0%) in 2020, whereas the DDDs of rosuvastatin increased from 598.8 million to 1563.2 million (+161.1%). The cost of atorvastatin decreased from 6662.0 million CNY to 2720.1 million CNY (−59.2%), whereas the expenditure for rosuvastatin decreased from 2738.6 million CNY to 1374.6 million CNY (−49.8%).

After policy implementation, both the DDDs and expenditures for Xuezhikang and Zhibitai increased. The DDDs of Xuezhikang increased from 29.3 million in 2019 to 50.6 million in 2020 (+82.9%); expenditure also increased from 143.7 million CNY to 264.6 million CNY (+84.1%). The increases in DDDs and expenditure for Zhibitai were relatively smaller than for Xuezhikang: DDDs grew from 8.1 to 12.6 million (+55.6%) and expenditure grew from 37.4 million CNY to 57.5 million CNY (53.7%).

Compared with Chinese patent medicines, the two chemical drugs still accounted for most of the market share. Before the centralized procurement policy, atorvastatin had the highest monthly shares of DDDs and expenditure at an average of 61.9% and 69.5%, respectively. After being included, the shares of DDDs and expenditure for atorvastatin decreased by −5.8% and −7.9%, respectively.

The DDDs and expenditures for rosuvastatin increased from 35.9% to 42.1% and from 28.6% to 31.1%, respectively, following policy implementation. No significant changes in the share of DDDs were observed for the Chinese patent medicines. Xuezhikang's expenditure share increased significantly (from 1.5% to 6.0%), indicating that centralized procurement policies did not significantly change drug use preferences for Chinese patent medicines.

Table 3 shows the results of applying the ITS model to the data shown in Figure 1. Before policy implementation, no significant trend was observed in atorvastatin's DDDs (*p* > 0.05) and procurement cost (*p* > 0.05). However, a significant increase of 72,419.7 thousand (*p* < 0.001) in atorvastatin DDDs and a significant decrease of 298.0 million CNY (*p* < 0.001) in expenditure for atorvastatin were observed in the month of policy implementation. No significant trend change was observed in the DDDs (*p* > 0.05) and cost (*p* > 0.05) of atorvastatin. Similarly, before policy implementation, no significant growth trend was observed in the DDDs (*p* > 0.05) and expenditure (*p* > 0.05) for rosuvastatin. However, the DDDs of rosuvastatin significantly increased by 73.0 million (*p* < 0.001) and expenditure decreased by 116.2 million CNY (*p* < 0.001) in the month of policy implementation. After policy implementation, no significant change was observed in the DDDs (*p* > 0.05) and expenditure (*p* > 0.05) for rosuvastatin. The policy had no significant effect on Xuezhikang's DDDs, which experienced an increase of 0.2 million (*p* < 0.01), whereas expenditure increased by 0.9 million CNY (*p* < 0.01). Furthermore, the policy had no significant effect on Zhibitai's DDDs (*p* > 0.05) and expenditure (*p* > 0.05). Before policy implementation, insignificant increases of 0.2 million (*p* < 0.05) and 0.9 million CNY (*p* < 0.05) were observed in Xuezhikang's DDDs and expenditure, respectively. Similarly, the DDDs (*p* > 0.05) and expenditure (*p* > 0.05) for Zhibitai did not exhibit significant growth trends before policy implementation.

**Table 3:** DDDs and expenditure for antihyperlipidemic drugs.

Drugs	DDDs (thousand)	Expenditure (million)
Atorvastatin		
Baseline trend	1136.5	6.164
Δ Intercept	72,419.7***	**−**298.0***
Δ Trend	732.1	**−**14.08
Constant	72,299.9***	528.7***
R Squared	0.889	0.825
DW	2.584	2.252
Rosuvastatin		
Baseline trend	1394.6	3.116
Δ Intercept	72,997.5***	**−**116.2***
Δ Trend	**−**619.1	**−**4.854
Constant	34,773.9***	213.8***
R Squared	0.952	0.781
DW	2.785	2.338
Xuezhikang		
Baseline trend	172.7*	0.851*
Δ Intercept	367.4	1.797
Δ Trend	**−**17.47	**−**0.0742
Constant	1162.7*	5.686*
R Squared	0.776	0.776
DW	1.760	1.758
Zhibitai		
Baseline trend	18.49	0.0853
Δ Intercept	**−**103.6	**−**0.293
Δ Trend	40.44	0.157
Constant	534.2***	2.453***
R Squared	0.755	0.737
DW	1.935	2.045

* *p* < 0.05

** *p* < 0.01

*** *p* < 0.001.

### 3.3 Heterogeneity Across Hospitals

**Figure 2:**
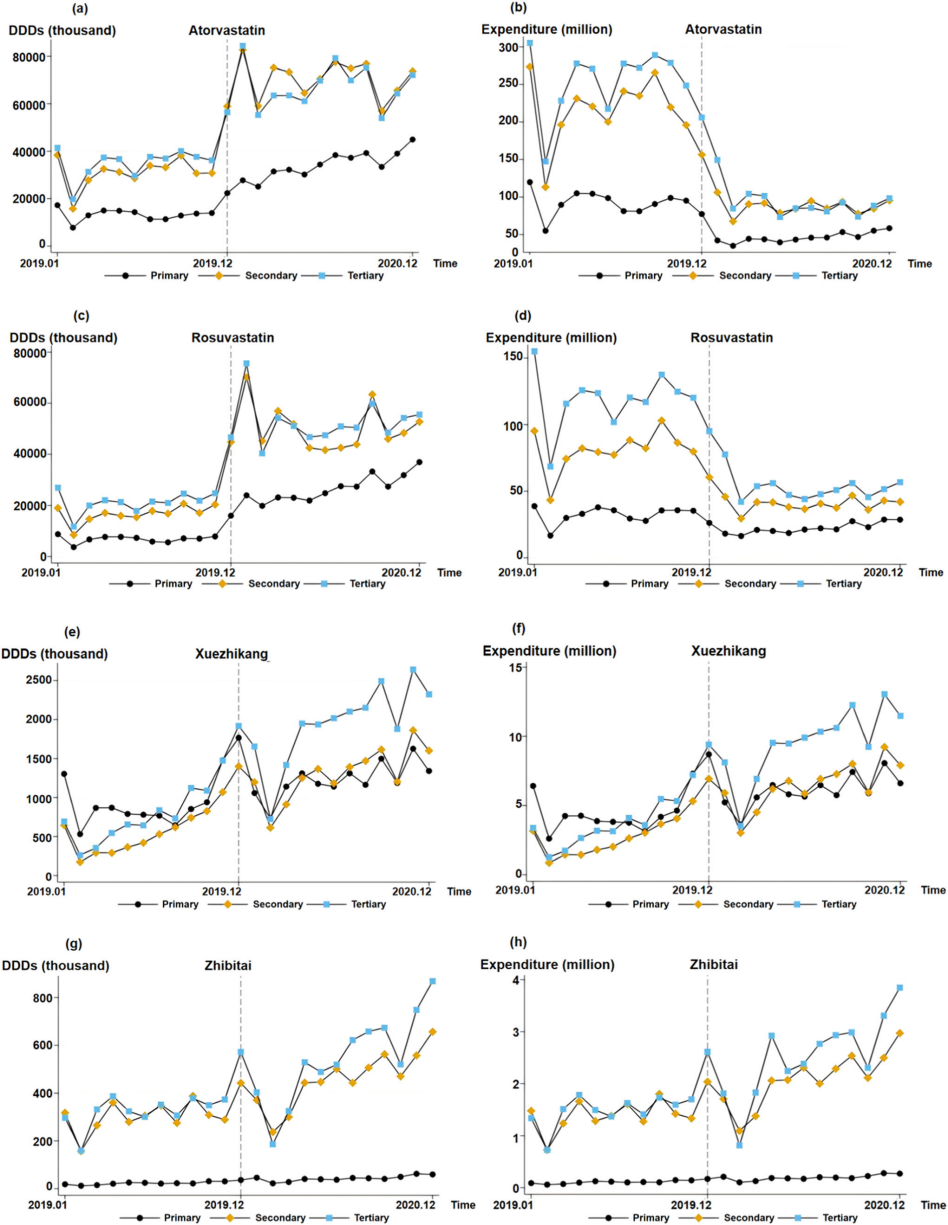
DDDs and expenditure of antihyperlipidemic drugs stratified by hospital level. (a) DDDs of atorvastatin stratified by hospital level. (b) Expenditure of atorvastatin stratified by hospital level. (c) DDDs of rosuvastatin stratified by hospital level. (d) Expenditure of rosuvastatin stratified by hospital level. (e) DDDs of Xuezhikang stratified by hospital level. (f) Expenditure of Xuezhikang stratified by hospital level. (g) DDDs of Zhibitai stratified by hospital level. (h) Expenditure of Zhibitai stratified by hospital level.

Figure 2 shows the DDDs and expenditures of atorvastatin, rosuvastatin, Xuezhikang, and Zhibitai in hospitals of different levels. The medication structures of rosuvastatin, atorvastatin, and Zhibitai were similar, with similar DDDs and expenditures in secondary and tertiary hospitals that were higher than those in primary hospitals. However, the DDDs of Zhibitai in primary hospitals were considerably lower than those in other hospitals. Before policy implementation, the DDDs of Xuezhikang were similar in hospitals of different levels. However, after policy implementation, the DDDs in tertiary hospitals gradually exceeded those in primary and secondary hospitals.

Table 4 applies the ITS to the four drugs in Figure 2 in hospitals of different levels. Before policy implementation, no significant growth trend was observed in the DDDs (*p* > 0.05) and expenditures (*p* > 0.05) of atorvastatin in hospitals of different levels. However, in the month of policy implementation, the DDDs of atorvastatin increased significantly by 10.2 million (*p* < 0.001), 35.8 million (*p* < 0.001), and 26.5 million (*p* < 0.001) in primary, secondary, and tertiary hospitals, respectively. In addition, expenditures decreased significantly by 44.9 million CNY (*p* < 0.01), 120.8 million CNY (*p* < 0.001), and 132.3 million CNY (*p* < 0.001) in primary, secondary, and tertiary hospitals, respectively. A significant growth of 1.5 million (*p* < 0.001) in the DDDs of atorvastatin was observed in primary hospitals and a significant decrease of 0.01 million CNY in spending was seen in tertiary hospitals (*p* < 0.05).

**Table 4:** DDDs and expenditure for antihyperlipidemic drugs stratified by hospital level.

Drugs	DDDs (thousand)			Expenditure (million)		
	Primary	Secondary	Tertiary	Primary	Secondary	Tertiary
Atorvastatin						
Baseline trend	**−**37.1	521.5	652.0	**−**0.139	2.486	3.815
Δ Intercept	10,224.3***	35,763.0***	26,499.6***	**−**44.9**	**−**120.8***	**−**132.3***
Δ Trend	1502.5***	**−**432.3	**−**355.0	0.3	**−**4.8	**−**9.7*
Constant	13,419.2***	27,838.5***	31,042.0***	93.5***	202.4***	232.8***
R Squared	0.951	0.889	0.819	0.736	0.808	0.841
DW	2.192	2.592	2.528	2.023	2.332	2.141
Rosuvastatin						
Baseline trend	37.3	553.1	382.2	0.517	1.732	0.867
Δ Intercept	9649.3***	32,697.1***	29,354.3***	**−**17.0***	**−**44.7***	**−**54.3**
Δ Trend	1284***	**−**854	**−**388	0.139	**−**2.222	**−**2.784
Constant	6599.7***	13,326.7**	18,908.3***	29.3***	70.6***	113.9***
R Squared	0.964	0.871	0.853	0.585	0.794	0.793
DW	2.574	2.517	2.944	2.172	2.502	2.188
Xuezhikang						
Baseline trend	18.12	62.79**	91.85**	0.09	0.31**	0.45**
Δ Intercept	166.8	92.3	108.3	0.823	0.463	0.512
Δ Trend	**−**1.60	**−**10.79	**−**5.09	**−**0.01	**−**0.05	**−**0.02
Constant	784***	166	213	3.843***	0.808	1.034
R Squared	0.381	0.815	0.853	0.386	0.815	0.854
DW	1.647	1.731	1.934	1.640	1.735	1.936
Zhibitai						
Baseline trend	1.432*	6.602	10.460	0.007*	0.030	0.049
Δ Intercept	**−**1.67	**−**31.92	**−**70.05	**−**0.009	**−**0.12	**−**0.16
Δ Trend	0.67	15.73	24.04	0.0026	0.066	0.088
Constant	13.8**	259.7***	260.7***	0.0661**	1.200***	1.187***
R Squared	0.816	0.735	0.730	0.801	0.713	0.705
DW	1.729	2.166	1.831	1.700	2.168	2.001

* *p* < 0.05

** *p* < 0.01

*** *p* < 0.001.

Before policy implementation, no significant growth trend was observed in the DDDs and expenditure for rosuvastatin in hospitals of different levels (*p* > 0.05). However, in the month of policy implementation, DDDs increased significantly by 9.7 million (*p* < 0.001) in primary hospitals, 32.7 million (*p* < 0.001) in secondary hospitals, and 29.4 million (*p* < 0.001) in tertiary hospitals. At the same time, costs decreased significantly by 17.1 million CNY (*p* < 0.001), 44.8 million CNY (*p* < 0.001), and 54.3 million CNY (*p* < 0.01) in primary, secondary, and tertiary hospitals, respectively. A significant growth trend (monthly growth of 1.3 million; *p* < 0.001) was observed in the DDDs of rosuvastatin in primary hospitals.

The policy had no significant effect on Zhibitai's DDDs (*p* > 0.05) or expenditures for Xuezhikang (*p* > 0.05). Before policy implementation, significant growth trends were observed for DDDs (a monthly increase of 0.06 million; *p* < 0.01) and expenditure (a monthly increase of 0.3 million CNY expenditure; *p* < 0.01) for Xuezhikang in secondary hospitals. A significant monthly increase of 0.09 million in DDDs (*p* < 0.01) and an overall increase of 0.5 million CNY in expenditure (*p* < 0.01) were observed in tertiary hospitals for Xuezhikang. For Zhibitai, a significant monthly increase of 0.01 million (*p* < 0.05) in DDDs and an overall increase of 0.007 million CNY in expenditure (*p* < 0.05) were observed in tertiary hospitals.

## 4 Discussion

In this study, we examined the effect of the expansion of the “4 + 7” pilot program on the utilization, costs, and affordability of chemical drugs (atorvastatin and rosuvastatin) and their corresponding Chinese patent medicine alternatives (Xuezhikang and Zhibitai) by investigating DDDs, expenditures, and affordability.

The study indicated that after policy implementation, the proportion of chemical drugs included in the clinical use of lipid‐lowering agents increased. The total amount of the two chemical drugs increased by 203.263 million DDDs (+126.5%). This phenomenon was closely related to the significant reduction in the prices of the bid‐winning lipid‐lowering drugs, indicating that the accessibility of the bid‐winning lipid‐lowering drugs was enhanced. In addition, the increase in DDDs may be associated with the need for hospitals to prioritize the use of selected products and fulfill their agreed procurement volumes under the purchase contract.

After policy implementation, the unit comparable expenditure of the two chemical drugs decreased significantly (by 363.7 million CNY), indicating that the policy delivered an initial cost‐saving effect on lipid‐lowering chemical drugs. Price is the primary determinant of drug affordability [14], and the unreasonable prices and high costs of lipid‐lowering drugs were important factors that restricted effective control of the drug burden for patients with hyperlipidemia in China [15]. In recent decades, the increase in ASCVD in the Chinese population has been significant, and only 14.5% of patients with ASCVD have received lipid‐lowering treatment [15]. Lower prices can effectively increase the treatment rate, thus generating long‐term benefits.

We observed that after policy implementation, the DDDs of lipid‐lowering Chinese patent medicines did not significantly decrease but increased by 28.77 million DDDs. These findings indicate that after policy intervention, the use of the two lipid‐lowering Chinese patent medicines was not significantly impacted, and no significant demand transfer between drugs occurred. The increase in the use of Chinese patent medicines may be attributable to multiple factors including product characteristics, patient choice, doctor prescribing habits, and policy guidance.

Concerning product characteristics, Chinese patent medicines are usually favored by patients for their unique efficacy and few side effects. Although multiple factors must be considered, the significance and reliability of drug efficacy remain the fundamental criteria that guide the choice of clinical medication. The widespread use of Chinese patent medicines is not only based on profound cultural traditions and theories of Chinese medicine but also on their scientifically proven efficacy. Modern research including randomized controlled trials and systematic evaluations continues to confirm the efficacy of Chinese patent medicines in specific indications. Among lipid‐lowering agents, some Chinese patent medicinal products have irreplaceable and differentiated competitive advantages and have high standards for rigidity and product viscosity. For example, systematic evaluations have shown that, compared with statin controls, Xuezhikang capsules reduce TC (total cholesterol) and LDL‐C levels while increasing HDL‐C (high‐density lipoprotein cholesterol) levels [16]. These Chinese patent medicinal products exhibit good lipid‐lowering effects, low rates of adverse reactions, and better tolerability, making them popular among doctors and patients. Numerous clinical studies with lipid‐lowering Chinese patent medicines including Xuezhikang and Zhibitai have also been conducted in China for the treatment of chronic diseases such as atherosclerosis, coronary heart disease, kidney disease, and hypertension [17]. These studies have highlighted benefits beyond cholesterol reduction, further promoting the clinical application of these agents. In addition, different target values are adopted for lipid‐lowering treatment in different atherosclerotic‐related diseases. Personalized lipid‐lowering treatment addresses differing needs with different lipid‐lowering agents that have varying efficacy, providing a role for the clinical application of lipid‐lowering Chinese patent medicine agents.

Patient choice is influenced by traditional Chinese medicine theories, personal physical condition, medication history, and emotional preferences. Older Chinese people have a high level of acceptance and recognition of Chinese medicines. Patients who have previously used Chinese patent medicines tend to maintain their choice of medication and do not leave the system [18]. In addition, factors such as an aging population, an increase in the rates of chronic disease morbidity, and an increase in public awareness of medical security are driving demand for Chinese patent medicines. Conducting relevant surveys at the patient level may further help elucidate factors such as treatment effectiveness, reasons for selection, and patient satisfaction with lipid‐lowering Chinese patent medicine agents.

The policy has not affected doctors' prescribing of Chinese patent medicines. Therefore, we presume that in clinical practice, doctors have not unreasonably increased the use of Chinese patent medicines to increase medical income following the reduction of the total cost of chemical drugs. With advanced research, Chinese patent medicines are becoming widely recognized in clinical practice, and their benefits are gradually being highlighted.

China has recently issued a series of encouraging policies to support the development of the traditional Chinese medicine industry. The implementation of long‐term comprehensive plans such as the “Outline Development Plan for the Development of Traditional Chinese Medicine (2016–2030)” and the “Healthy China 2030” plan is a long‐term driving force for the development of the traditional Chinese medicine industry and promotes joint efforts of pharmaceutical companies and medical institutions that are beneficial to the Chinese patent medicine market.

Moreover, ITS results indicated no significant upward trend in DDDs and expenditure for Chinese patent medicines after policy implementation. By analyzing the changes in DDDs and expenditure in specific areas, significant differences in DDDs and expenditure for lipid‐lowering drugs were observed between primary, secondary, and tertiary hospitals before policy implementation. DDDs and expenditures in secondary and tertiary hospitals were considerably higher than those in primary hospitals. We presume that this phenomenon is related to the replacement effect of Xuezhikang. The recognition, drug availability, and affordability of Xuezhikang were superior to those of Zhibitai capsules in grass‐roots areas. No significant changes in trend were observed across hospital levels for lipid‐lowering drugs such as Zhibitai and Xuezhikang following policy implementation (*p* > 0.05), indicating that the substitution effect of bid‐winning chemical drugs had no impact on Chinese patent medicines at all levels of medical institutions.

In summary, NVBP achieved an initial cost‐saving effect in the short term through the inclusion of high‐quality and widely used types of lipid‐lowering drugs, reflecting China's drive toward the fairness and accessibility of pharmaceutical services. In the future, further expanding the scope of procurement to include chronic diseases and drugs with high demand in the procurement catalog is expected to effectively reduce the drug burden of Chinese patients.

Future scholarly endeavors should concentrate on examining China's paradigm shift from a volume‐centric to a value‐centric approach to pharmaceutical procurement [19]. The success of NVBP should not be measured solely by price or procurement volume but should be based more on its contribution to improving health outcomes and guaranteeing access to essential medicines [20]. Chemical drug procurement has little impact on innovative drugs, providing opportunities for the development of innovative drugs for the proprietary Chinese patent medicine industry. Under the policy, enterprises can increase research and development investment to develop innovative drugs with characteristics that meet the market demand for innovative drugs. Meanwhile, in its promotion of the procurement of Chinese medicines, the government should consider the special characteristics of these medicines and formulate differentiated procurement rules—such as giving special consideration to exclusive and protected Chinese medicines—to promote the healthy development of the industry.

The study has some limitations. First, the study results are based on drug purchase data rather than drug use data (such as prescriptions). Despite the strong consistency between purchase and use data, a possibility remains that the two data sources do not fully match one another. Second, ITS models were constructed based on policy intervention before and after 24 months of data. This process involved examining several points during and after policy interventions, which may limit understanding of the long‐term impact of volume procurement of lipid‐lowering chemical drugs on Chinese patent medicines. Third, the study used atorvastatin and rosuvastatin as representatives of chemical drugs and Xuezhikang and Zhibitai as representatives of Chinese patent medicines. Although these medicines occupy a relatively large market share, this practice may nevertheless have affected the generalizability of the conclusion. Finally, the price of Chinese patent medicines is influenced by a variety of factors—such as raw material availability—that cause large fluctuations that can counteract some price trends.

## 5 Conclusion

This study analyzed the impact of NVBP on lipid‐lowering drugs before and after policy implementation using 24 months of data. The results demonstrated that the procurement of chemical drugs did not cause overall changes in the use of Chinese patent medicines. By analyzing the classification and analysis of lipid‐lowering chemical drugs at the national level in terms of selected and nonselected varieties, originator drugs, generic drugs, and non‐approved varieties, we observed that “4 + 7” expanded procurement had a significant impact on lipid‐lowering chemical drugs. The DDDs and expenditure for Chinese patent medicines that were not covered by the policy did not decline, indicating that the volume procurement of chemical drugs significantly reduced the price of drugs and increased affordability. However, overall, doctors did not reduce their use of Chinese patent medicines, and no obvious substitution between these medicines and chemical medicines was observed, indicating the specificity of Chinese patent medicines in clinical use.

In addition, the policy did not cause a decline in the use of Chinese patent medicines. The analysis of changes in DDDs and expenditures across different levels of hospitals showed that the structure of the DDDs of Chinese patent medicines has not been affected. We further speculated that irrational medication behavior—such as reducing the total cost of chemical drugs by lowering the price and prescribing more Chinese patent medicines—did not occur.

Chemical drugs have a clear composition, controllable quality, a well‐defined mechanism of action, and demonstrated clinical efficacy. However, Chinese patent medicines have the advantage of treating some major chronic diseases before they are diagnosed. Clinicians can benefit from combining Chinese patent medicines and chemical drugs to improve treatment efficacy while reducing drug toxicity. Therefore, chemical drugs and Chinese patent medicines should be used rationally in clinical practice based on their characteristics.

## Author Contributions

**Zhao Yang:** conceptualization (equal), data curation (equal), formal analysis (equal), funding acquisition (equal), project administration (equal), writing – original draft (equal), writing – review and editing (equal). **Xiao Han:** data curation (equal), formal analysis (equal), investigation (equal), methodology (equal), writing – original draft (equal). **Pei Liang:** formal analysis (equal), investigation (equal), methodology (equal), visualization (equal), writing – original draft (equal), writing – review and editing (equal). **Xiaoting Zhao:** formal analysis (equal), methodology (equal), validation (equal), writing – original draft (equal), writing – review and editing (equal). **Qiyun Zhu:** formal analysis (equal), software (equal), writing – original draft (equal), writing – review and editing (equal). **Hui Ye:** conceptualization (equal), resources (equal), supervision (equal), writing – original draft (equal), writing – review and editing (equal). **Chao Yang:** conceptualization (equal), project administration (equal), supervision (equal), writing – review and editing (equal). **Bin Jiang:** conceptualization (equal), funding acquisition (equal), methodology (equal), project administration (equal), writing – review and editing (equal).

## Ethics Statement

The authors have nothing to report.

## Consent

The authors have nothing to report.

## Conflicts of Interest

The authors declare no conflicts of interest.

## Data Availability

Restrictions apply to the availability of these data, which were used under license for this study.

## References

[1] T. Vos , S. S. Lim , C. Abbafati , et al., “Global Burden of 369 Diseases and Injuries in 204 Countries and Territories, 1990–2019: A Systematic Analysis for the Global Burden of Disease Study 2019,” Lancet 396, no. 10258 (2020): 1204–1222, 10.1016/S0140-6736(20)30925-9. 33069326 PMC7567026

[2] D. Zhao , J. Liu , M. Wang , X. Zhang , and M. Zhou , “Epidemiology of Cardiovascular Disease in China: Current Features and Implications,” Nature Reviews Cardiology 16, no. 4 (2018): 203–212, 10.1038/s41569-018-0119-4. 30467329

[3] J. Borén , M. J. Chapman , R. M. Krauss , et al., “Low‐Density Lipoproteins Cause Atherosclerotic Cardiovascular Disease: Pathophysiological, Genetic, and Therapeutic Insights: A Consensus Statement From the European Atherosclerosis Society Consensus Panel,” European Heart Journal 41, no. 24 (2020): 2313–2330, 10.1093/eurheartj/ehz962. 32052833 PMC7308544

[4] C. Baigent , A. Keech , P. M. Kearney , et al., “Efficacy and Safety of Cholesterol‐Lowering Treatment: Prospective Meta‐Analysis of Data From 90,056 Participants in 14 Randomised Trials of Statins,” Lancet 366, no. 9493 (2005): 1267–1278, 10.1016/S0140-6736(05)67394-1. 16214597

[5] Z. Wang , J. Liu , J. Li , et al., “Chinese Guidelines for Lipid Management (2023),” Chinese Circulation Journal (in Chinese) 38, no. 3 (2023): 237–271.

[6] C. Wang , W. Pang , X. Du , et al., “Efficacy and Safety of Zhibitai in the Treatment of Hyperlipidemia: A Systematic Review and Meta‐Analysis,” Frontiers in Pharmacology 13 (2022): 974995, 10.3389/fphar.2022.974995. 36120312 PMC9479062

[7] L. Guang‐Run , L. Li‐Hong , L. Ya‐li , and G. Qing , “Comparison of the Efficacy and Safety of Xuezhikang and Simvastatin the Treatment of Primary Hyperlipidemia in Chinese Adults,” Chinese Journal of Clinical Pharmacology (in Chinese) 37, no. 3 (2021): 305–307.

[8] Federation China Heart, “Chinese Expert Consensus on the Use of Zhibitai Capsules,” *Zhonghua Nei Ke Za Zhi (in Chinese)* 56, no. 8 (2017): 628–632. 10.3760/cma.j.issn.0578-1426.2017.08.016 28789500

[9] Y. Gao , X. Chen , C. Li , H. Wang , J. Tian , and F. Fu , “Toxicological Evaluation of, Red Rice Yeast Extract, Xuezhikang: Acute, 26‐Week Chronic and Genotoxicity Studies,” Regulatory Toxicology and Pharmacology 114 (2020): 104654, 10.1016/j.yrtph.2020.104654. 32278069

[10] C. E. C. G. Capsules , “Chinese Expert Consensus on the Clinical Application of Xuezhikang Capsules,” Chinese Community Doctors (in Chinese) 25, no. 14 (2009): 9–10.

[11] V. J. Wirtz , W. A. Kaplan , G. F. Kwan , and R. O. Laing , “Access to Medications for Cardiovascular Diseases in Low‐ and Middle‐Income Countries,” Circulation 133, no. 21 (2016): 2076–2085, 10.1161/CIRCULATIONAHA.115.008722. 27217433 PMC4880457

[12] P. K. S. Raju , “WHO/HAI Methodology for Measuring Medicine Prices, Availability and Affordability, and Price Components,” in Medicine Price Surveys, Analyses and Comparisons, ed. S. Vogler (Academic Press, 2019), 209–228.

[13] J. Yuan , Z. K. Lu , X. Xiong , and B. Jiang , “Lowering Drug Prices and Enhancing Pharmaceutical Affordability: An Analysis of the National Volume‐Based Procurement (NVBP) Effect in China,” BMJ Global Health 6, no. 9 (2021): e005519, 10.1136/bmjgh-2021-005519. PMC8438819 34518200

[14] National Academies of Sciences EAM, Division HAM, Services BOHC, Therapies COEP , Making Medicines Affordable: A National Imperative (National Academies Press (US), 2017). 29620830

[15] M. Zhang , Q. Deng , L. Wang , et al., “Prevalence of Dyslipidemia and Achievement of Low‐Density Lipoprotein Cholesterol Targets in Chinese Adults: A Nationally Representative Survey of 163, 641 Adults,” International Journal of Cardiology 260 (2018): 196–203, 10.1016/j.ijcard.2017.12.069. 29622441

[16] M. Li , Z. Han , W. Bei , X. Rong , J. Guo , and X. Hu , “Oleanolic Acid Attenuates Insulin Resistance via NF‐κB to Regulate the IRS1‐GLUT4 Pathway in HepG2 Cells,” Evidence‐Based Complementary and Alternative Medicine 2015, no. 1 (2015): 468520, 10.1155/2015/468520. 26843885 PMC4710921

[17] Z. Sidao , Y. Cui , and Z. Chengying , “Meta‐Analysis of Xuezhikang Capsules in the Treatment of Patients With Dyslipidemia,” China Medical Herald 16, no. 14 (2019): 51–55.

[18] M. Cao , “Industry Based on 'Diamond' Model Countermeasures of Chinese Traditional Patent Medicine Research on Competitiveness and Development [Master],” Chengdu University of Traditional Chinese Medicine (in Chinese) (2020).

[19] T. T. Chee , A. M. Ryan , J. H. Wasfy , and W. B. Borden , “Current State of Value‐Based Purchasing Programs,” Circulation 133, no. 22 (2016): 2197–2205, 10.1161/circulationaha.115.010268. 27245648 PMC5378385

[20] Z. Zhu , Q. Wang , Q. Sun , J. Lexchin , and L. Yang , “Improving Access to Medicines and Beyond: The National Volume‐Based Procurement Policy in China,” BMJ Global Health 8, no. 7 (2023): e011535, 10.1136/bmjgh-2022-011535. PMC10577736 37463786

